# Environmental Factors Affecting Freshwater Snail Intermediate Hosts in Shenzhen and Adjacent Region, South China

**DOI:** 10.3390/tropicalmed7120426

**Published:** 2022-12-09

**Authors:** Fengyang Min, Jiasheng Wang, Xiaoguang Liu, Yi Yuan, Yunhai Guo, Kongxian Zhu, Zhaohui Chai, Yunchao Zhang, Shizhu Li

**Affiliations:** 1 Key Laboratory of Parasite and Vector Biology, Ministry of Health, National Institute of Parasitic Diseases, Chinese Center for Disease Control and Prevention, National Center for Tropical Diseases Research, WHO Collaborating Center for Tropical Diseases, Shanghai 200025, China; 2 Department of River Research, Changjiang River Scientific Research Institute, Wuhan 430070, China; 3 Hubei Provincial Center for Disease Control and Prevention, Wuhan 430079, China

**Keywords:** freshwater snails, environmental factors, decision trees, predators, parasitic diseases, human activities

## Abstract

Sound knowledge of the local distribution and diversity of freshwater snail intermediate hosts and the factors driving the occurrence and abundance of them is crucial to understanding snail-borne parasitic disease transmission and to setting up effective interventions in endemic areas. In this study, we investigated the freshwater snails, water quality parameters, physical characteristics of habitats, predators and competitors, and human activity variables at 102 sites during December 2018 and August 2019 in Shenzhen and adjacent areas in China. We used decision tree models and canonical correspondence analysis to identify the main environmental and biotic factors affecting the occurrence and abundance of snail species. A total of nine species of snail were collected throughout the study area, with *Biomphalaria straminea*, *Sinotaia quadrata*, and *Physella acuta* being the most predominant species. Our study showed that the most important variables affecting the abundance and occurrence of snail species were the presence of predators and competitors, macrophyte cover, chlorophyll-*a*, substrate type, river depth, and water velocity. In terms of human activities, snail species occurred more frequently and in larger numbers in water bodies affected by human disturbances, especially for sewage discharge, which may reduce the occurrence and abundance of snail predators and competitors. These findings suggest that proper management of water bodies to reduce water pollution may increase the abundance of snail predators and competitors, and should be considered in integrated snail control strategies in the study area.

## 1. Introduction

Snails are invertebrate animals of the class Gastropoda and are widely distributed in aquatic ecosystems around the world. Approximately 5000 species have been identified in freshwater habitats such as lakes, rivers, streams, ponds, and dams [1,2]. Among these, some freshwater snails have medical and veterinary health importance, serving as vectors of parasitic diseases. Snail-borne diseases are major parasitic diseases that remain important public health issues worldwide, particularly in impoverished countries [3]. Schistosomiasis is an endemic parasitic disease affecting almost 240 million people worldwide, and an additional 700 million people are at risk of infection [4]. Six species of the blood fluke are reported to infect humans, causing schistosomiasis; among these, *Schistosoma haematobium*, *Schistosoma mansoni*, and *Schistosoma japonicum* are the main pathogenic species. Schistosoma eggs are the main pathogenic factors of schistosomiasis; parasitizing on host tissues, they cause the host to develop immunopathological reactions, which lead to the occurrence of urinary and reproductive system inflammation (*Schistosoma haematobium*) and obstructive diseases or intestinal diseases, liver and spleen inflammation, and liver fibrosis (*Schistosoma mansoni* and *Schistosoma japonicum*) [5]. In China, *schistosomiasis japonicum* is still prevalent in Hubei, Hunan, Jiangxi, Anhui, Jiangsu, Sichuan, and Yunnan provinces, posing a great threat to social and economic development [6]. Angiostrongyliasis cantonensis is another parasitic disease endemic in many areas, including Southeast Asia, the Pacific Islands, parts of South and Central America, and the Caribbean [7,8]. It is a serious disease with eosinophilic encephalitis and meningoencephalitis as the main clinical manifestations [7,9]. By 2012, more than 3000 cases of Angiostrongyliasis cantonensis had been recorded in nearly 300 countries and regions, of which the main outbreaks occurred in endemic areas, especially in China [10]. For example, 160 cases occurred in 2006 in Beijing, six cases occurred in 2007 in Guangdong, and 35 cases occurred from 2007 to 2008 in Yunnan; these intensive infections have aroused great attention among the public [11,12,13].

The distribution of snail-borne diseases largely depends on the spatial distribution of intermediate hosts [14]. It has been proven that snail-borne parasitic disease is endemic in areas where intermediate host snails are identified, while it does not occur in areas without host snails, although imported parasitic disease cases have been detected [15]. Snail distribution and abundance generally depend on various environmental factors, including physical factors such as temperature, precipitation, aquatic macrophyte cover, hydrography, and substrate composition; chemical factors such as pH, electrical conductivity, five-day biochemical oxygen demand (BOD_5_), chemical oxygen demand, total nitrogen, and total phosphorus; and biological factors such as food, competition, and predator–prey interactions [16,17,18]. However, the relative importance of environmental factors varies considerably in different regions due to the environmental heterogeneity [19], indicating that local surveys are needed to determine the preferred habitats of snail hosts.

A better understanding of the environmental factors affecting the distribution and habitat preferences of snail intermediate hosts is crucial for the effective control and elimination of snail-borne diseases. In Shenzhen and adjacent areas, a few studies have been conducted on the biology of several freshwater snails [20,21,22,23,24,25]. However, the sample sites are very scattered, and the surveyed snails are mainly *Biomphalaria straminea* and *Pomacea canaliculata*. Little is known about the distribution of the snails and the main factors affecting the snail abundance in the region, which is unfavorable for promoting comprehensive prevention and control measures for snail-borne diseases. Therefore, in this study, we aimed to (i) identify the local distribution and diversity of freshwater snail intermediate hosts of parasites, and (ii) to identify the biotic and abiotic factors that affect the occurrence and abundance of these snails in Southern China. The findings of this study could be helpful for priority habitat identification and to obtain targets for the prevention and control of snail-borne diseases in this area.

## 2. Materials and Methods

### 2.1. Study Area

**Figure 1:**
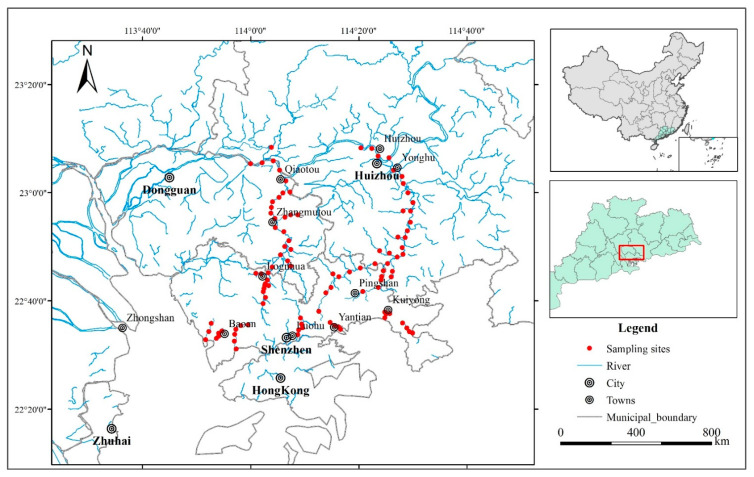
The study area and sampled sites in Shenzhen and adjacent areas, South China.

The study was conducted in the rivers of Shenzhen and the adjacent waters of Dongguan and Huizhou, which are located between latitudes 22°30′54.34″ N and 23°13′53.64″ N and longitudes 113°48′6.77″ E and 114°30′40.26″ E (Figure 1). This area is a subtropical monsoon climate domain, featuring hot and humid summers and mild winters. The averages of annual temperature and annual precipitation are approximately 23.0 °C and 1800 mm/m^2^, respectively. The precipitation in this area has great seasonal fluctuations, with 96% of rainfall concentrated during the wet season (April to September) and 4% of rainfall in the dry season (October to March) [26]. During recent decades, this area has been subjected to considerable human pressures, which mainly originate from rapid human population growth and enormous urbanization and development. Due to the rapidly developing economy, intensive human activities such as levee construction, pollution discharge, and dredging represent great anthropogenic stressors to the river ecosystems [27].

### 2.2. Malacological Surveys

Malacological surveys were conducted during December 2018 (dry season) and August 2019 (wet season) and were undertaken at 102 sites in the river systems of Shenzhen, Dongguan, and Huizhou cities (Figure 1). Sampling was undertaken by two experienced field collectors using snail scoops. The scoops were composed of a wire mesh measuring 1.5 × 1.5 mm, supported on an iron frame (40 × 30 cm), and mounted on a 1.5-m-long iron handle [28]. At each site, the investigators collected all snails found in a radius of approximately 2 m over a permitted search time of half an hour. Collected snails were transferred to plastic vials containing 10% formaldehyde and transported in plastic containers to the laboratory of the Hubei Provincial Center for Disease Control and Prevention for processing. Snails were identified to species level based on shell morphological characteristics following the published identification guidelines [29] and descriptions from previous reports on snail sightings in the region [30,31]. Information on the 102 sites are listed in Table S1.

### 2.3. Local Environmental Variables

Physico-chemical water quality measurements were performed both onsite during sampling and in the laboratory. Water temperature (WT), pH, electrical conductivity (Cond), dissolved oxygen (DO), and total dissolved solids (TDS) were measured in the field using a multi-parameter probe (YSI V6620; YSI Company, Yellow Springs, OH, USA). In addition, water samples were collected from each sampling site in polyethylene bottles and transported (in the dark) to the laboratory in an ice-cooled box for other physico-chemical parameter analyses. Total nitrogen (TN), ammonia (NH_4_-N), nitrate (NO_3_-N), total phosphorus (TP), orthophosphate (PO_4_-P), chlorophyll-*a* (Chl-a), and chemical oxygen demand (COD) were determined according to the Chinese standard method [32] at the laboratory of the Changjiang River Scientific Research Institute. Water depth was measured using a graduated stick calibrated in centimeters. River width, emergent macrophyte width, and floating macrophyte width were determined with a rangefinder. Water velocity was measured using a flowmeter (FP111, Global Water, Sunnyvale, CA, USA). At each sampling site, the substrate was carefully assessed by observation and classified as silt, sand, gravel, pebbles, cobbles, or boulders [33]. Taking the sampling point as the center, we visually estimated the coverage ratio of aquatic plants (emergent, submerged, and floating) by a simple estimation of the coverage ratio of aquatic plants within 500 m. The percentage of aquatic macrophyte cover was classified into four groups: very low (<10%), low (10–35%), moderate (35–65%), and high (>65%) [34].

### 2.4. Predator and Competitor Survey

Macroinvertebrates were taken from the substrate with a weighted Petersen grab (0.0625 m^2^) and then passed through a 420 mm sieve. Specimens were manually sorted from sediment on a white porcelain plate and preserved in 10% formalin and then sent to the laboratory for family-level identification according to relevant references [29,35,36,37,38,39]. The collected macroinvertebrates were assigned to functional feeding groups: predators, scrapers, gatherer–collectors, filterer–collectors, and shredders [37,40]. Scrapers and macroinvertebrates belonging to the family Physidae were considered competitors of snails [41]. The invertebrates, such as Dytiscidae beetles (Insecta: Coleoptera) [42], Belostomatidae bugs (Insecta: Hemiptera) [43], Odonates (Insecta: Odonate) [44], Psychodidae (Insecta: Diptera) [45], Hydrophilidae (Insecta: Coleoptera) [46], and Glossiphoniidae leeches (Hirudinea: Rhynchobdellida) [47], were considered snail predators.

### 2.5. Human Disturbance

Human activities at each surveyed site, such as fishing, shipping, clothes washing, dredging, pollution discharge, farming, and irrigation, were classified by their average disturbance intensity around the area [48]. A score of 1 was awarded for minimal disturbance, 2 for medium disturbance, and 3 for high disturbance.

### 2.6. Data Analysis

#### 2.6.1. Classification and Regression Tree (CART)

Thirty-two environmental variables (both biotic and abiotic factors) were used to predict the occurrence and abundance of snail species (Table 1). Classification and regression trees (CART) were applied to develop the models. The models tend to predict that the most common species will always be present and the rarest will always be absent [49]. Thus, the frequency of occurrence of snail species of more than 20% was included in the prediction model, as suggested by Yigezu et al. [49] Based on a training set of 102 samples, classification and regression trees were used to develop the models. The classification tree models were built using Weka (version 3.8.5, University of Waikato, New Zealand), applying the J48 algorithm. The J48 algorithm is a Java re-implementation of C4.5, which is well known and has been frequently used over the years [50,51]. The algorithms for the induction of decision trees are based on the top-down induction of decision trees (TDIDT) principle [52]. Likewise, regression tree models were built using Weka and applying the M5 algorithm to relate the abundance of snail species and environmental variables. M5 is a very popular regression tree algorithm; it splits the entire dataset into smaller subsets via the divide and conquer method. This procedure reduces the parameter space into sections (subspaces) and develops a linear regression model in every one of them [53]. Default parameter settings were used to induce the trees, and all of the models were subjected to 10-fold cross-validation [54].

The percentage of correctly classified instances (CCI, %) [54] and Cohen’s kappa statistics (K) [55] were used to evaluate the performance of the classification trees. CCI is calculated as the percentage of the true predictions; Cohen’s kappa statistic simply measures the proportion of all possible cases of presence or absence that are predicted correctly by a model after accounting for chance predictions. Models with a CCI higher than or equal to 70% and K higher than or equal to 0.4 were considered reliable [56,57,58], while according to Landis and Koch [59], we determined the degree of agreement when Cohen’s kappa was found to be in various ranges, such as ≤0 (poor); 0–0.2 (slight); 0.2–0.4 (fair); 0.4–0.6 (moderate); 0.6–0.8 (substantial), and 0.8–1 (almost perfect).

#### 2.6.2. Canonical Correspondence Analysis (CCA)

To elucidate the relationships between different snail species and environmental variables, a canonical correspondence analysis (CCA) was performed. The CCA was restricted to those sites with complete data records and where at least one snail species existed (n = 70). The forward selection method was used to screen environmental factors, and the Monte Carlo method was used to test the significance of environmental variables (999 unrestricted permutations). The CCA was conducted in the R software package, version 3.6.2 (R Foundation for Statistical Computing; Vienna, Austria), using the package “vegan” [60].

## 3. Results

### 3.1. Environmental Conditions

In general, there were some differences in water quality between different sampling periods. The physical habitat conditions, such as water temperature, river depth, and water velocity, were significantly different between the wet and dry seasons; however, the river width and substrate type did not differ between the wet and dry seasons. According to the Chinese standard “Environmental quality standards for surface water (GB3838-2002)”, the water quality of the sampling sites in the dry season was in the inferior V class, and 69.2% of the sampling sites’ water quality in the wet season was in the inferior V class. The main over-standard items were total nitrogen (TN) and total phosphorus (TP). The maximum total nitrogen (TN) and total phosphorus (TP) contents were up to 27.50 mg/L and 2.97 mg/L, respectively, which are 13.3 times and 7.4 times the value corresponding to each Class V water quality standard.

### 3.2. Occurrence and Abundance of Freshwater Snails

A total of 5238 freshwater snails were collected from 102 different sampling sites. The snails were encountered in 70 sampling sites (68.6%). Collected snails were found belonging to six families (Viviparidae, Physidae, Lymnaeidae, Planorbidae, Semisulcospiridae, and Ampullariidae) and nine species (Table 2); all of them are intermediate hosts of parasites. The nine species included *Sinotaia quadrata*, *Cipangopaludina chinensis*, and *Physella acuta* (all of which are intermediate hosts of *Angiostrongylus cantonensis*); *Sinotaia limnophila* (the intermediate host of *Echinostoma revolutum)*; *Radix auricularia* (intermediate host of *Angiostrongylus cantonensis* and *Fasciola hepatica*); *Biomphalaria straminea* (the intermediate host of *Schistosoma mansoni* and *Angiostrongylus cantonensis*); *Semisulcospira cancellata* (the intermediate host of *Angiostrongylus cantonensis*, *Clonorchis sinensis*, and *Paragonimus westermani*); *Semisulcospira libertina* (the intermediate host of *Paragonimus westermani*), and *Pomacea canaliculata* (the intermediate host of *Angiostrongylus cantonensis*, *Echinostoma revolutum*, and *Gonathostoma spinigerum*).

Among all genera of snail species collected, *B. straminea* was the predominant snail species, accounting for 54.7% of the total number of snails, and it was encountered in 31.4% of the sampling sites. *Sinotaia quadrata* was the second most common snail species, accounting for 13.0% of collected snails, and occurred in 25.5% of the surveyed sites. Although *P. acuta* accounted for only 8.6% of the snail population, it was found with a frequency of occurrence of 22.5% in all surveyed sites. The least common snail species were *P. canaliculata*, *S. limnophila,* and *C. chinensis*, which were found in <10% of the sampling sites (Table 3).

### 3.3. CART Analysis

#### 3.3.1. Variables of Importance

**Figure 2:**
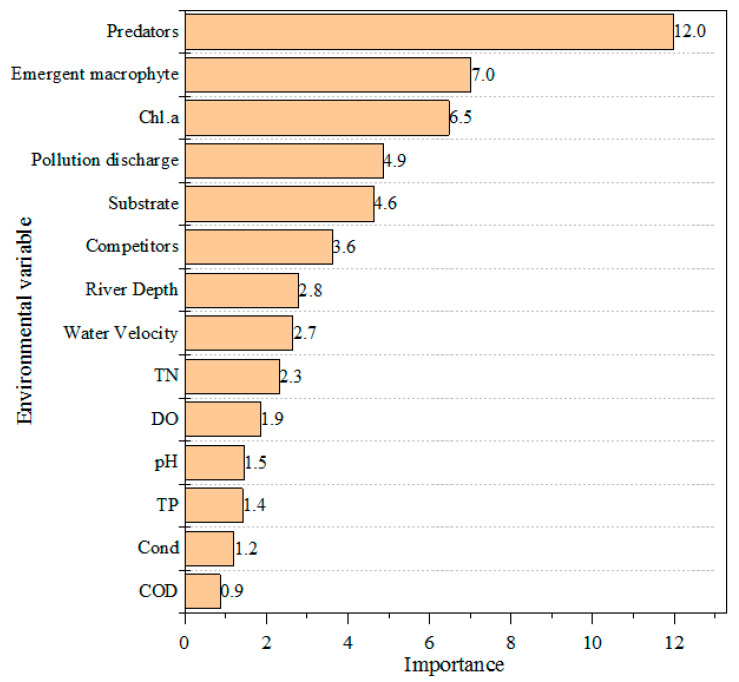
Overview of importance of the different input variables used in the decision tree models to model the presence or absence of each freshwater snail species.

Thirty-two environmental variables were applied to predict the presence/absence of snail species whose frequency of occurrence was higher than 20% (Table 3); these are *B. straminea*, *S. quadrata* and *P. acuta.* From the induced process of decision tree construction, we were able to obtain the importance of different variables of the three species (Figures S1–S3). Thus, the comprehensive variable importance could be obtained from the arithmetic means of these three species (Figure 2). According to Figure 2, the most important environmental variables were predators’ occurrence (12.0), emergent macrophyte cover (7.0), and chlorophyll-*a* (6.5). In addition, the importance values for pollution discharge, substrate type, competitors’ occurrence, river depth, and water velocity in the classification tree models were 4.9, 4.6, 3.6, 2.8, and 2.7, respectively. However, some of the chemical water quality variables’ importance values, such as those for DO, pH, TP, Cond, and COD, were less than 2.0 and thus they were less critical for explaining the occurrence of snail species.

**Figure 3:**
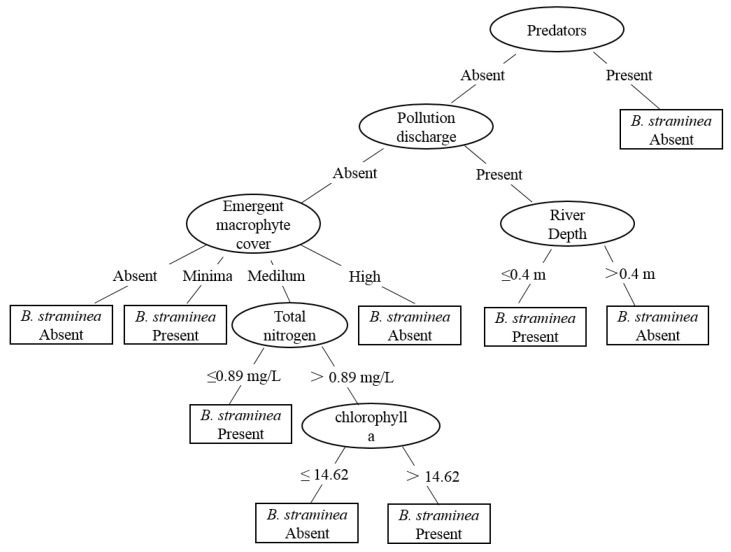
Classification tree model predicting the presence or absence of *Biomphalaria straminea*.

The classification tree model for *B. straminea* is shown in Figure 3. This tree has nine leaves and fifteen branches. The classification tree selected predators’ appearance as the root of the tree, which is considered the most important variable by which to predict the occurrence of *B. straminea*. That is suggested *B. straminea* was absent in the presence of predators. In addition, *B. straminea* was often present in sites where there was lower emergent canopy cover, shallow water habitats, and high chlorophyll-*a* levels (>14.62 μg/L).

**Figure 4:**
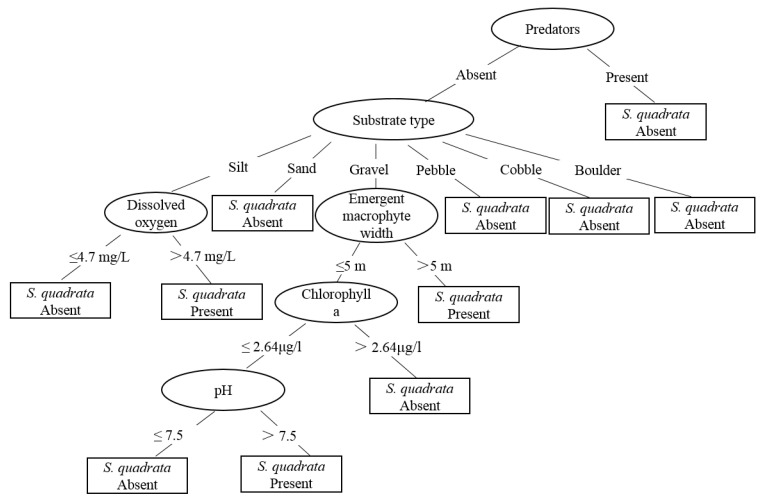
Classification tree model predicting the presence or absence of *Sinotaia quadrata*.

Figure 4 shows the classification tree model information for *S. quadrata*. The tree has eleven leaves and seventeen branches. The emergence of *S. quadrata* was mainly affected by the absence of predators. *Sinotaia quadrata* was usually found in environments with high oxygen concentrations (>4.7 mg/L). In addition, *S. quadrata* favored wide emergent macrophyte values (>5 m) and an alkaline environment (pH > 7.5).

**Figure 5:**
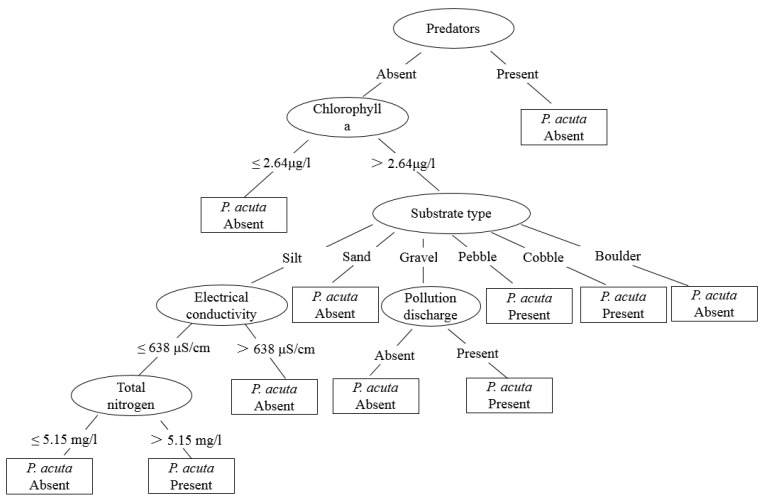
Classification tree model predicting the presence or absence of *Physella acuta*.

The classification tree model for *P. acuta* has eleven leaves and seventeen branches (Figure 5). Here, predators’ appearance was selected as the root of the tree and was considered the most important variable to predict the occurrence of this snail. *Physella acuta* prefer to inhabit a coarse-grained benthic environment. However, high conductivity and concentration of total nitrogen, in addition to pollution discharge environment, were preferable regarding the occurrence of *P. acuta*.

#### 3.3.2. Model Performance Evaluation

**Figure 6:**
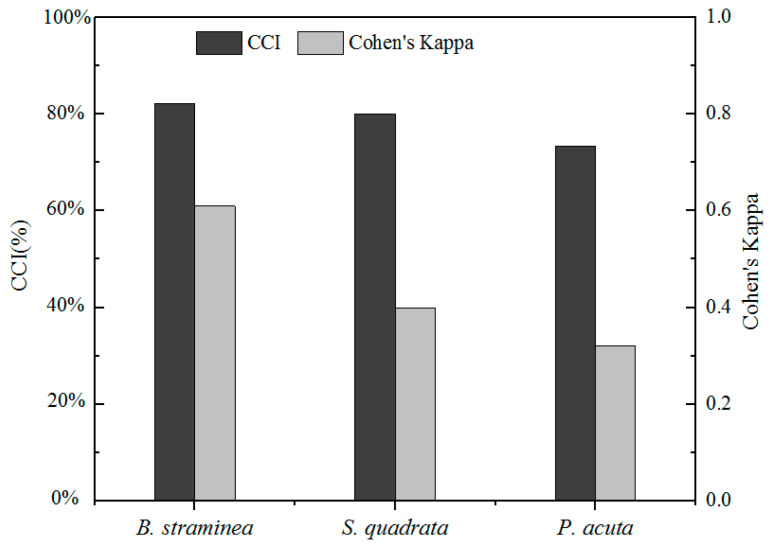
Overview of the average predictive performance of each classification tree model and the variation in the model based on CCI and Cohen’s kappa.

The model performance based on the CCI and Cohen’s kappa statistic (K) for the three species of snails is presented in Figure 6. According to the CCI, *B. straminea*, *S. quadrata,* and *P. acuta* had very good predictions (CCI > 70%). Among these, *B. straminea* had the highest model predictive performance, with a CCI value of 78%. However, *B. straminea* and *S. quadrata* were predicted accurately based on Cohen’s kappa statistic (K ≥ 0.4), and *P. acuta* had fair predictive performance with K = 0.32.

### 3.4. CCA Analysis

**Figure 7:**
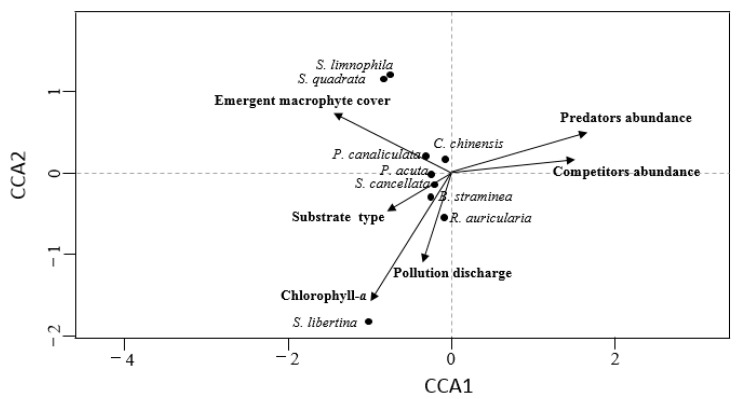
Canonical correspondence analysis ordination plot of snail species and predictor variables.

Selected variables found to be relevant for the CCA were the predator and competitor abundance, emergent macrophyte cover, chlorophyll-*a*, pollution discharge, and substrate type (Figure 7). The first axis was positively correlated mainly with predator and competitor abundance, but negatively correlated with emergent macrophyte cover. The second axis was negatively correlated mainly with chlorophyll-*a* and pollution discharge. According to Figure 7, most of the snail species were negatively correlated with predators and competitors. However, *P. canaliculata* was positively correlated with emergent macrophyte cover and *S. libertina* was positively correlated with chlorophyll-*a*.

## 4. Discussion

A fundamental understanding of the ecology of snail intermediate hosts is essential to plan and implement effective snail-borne disease control strategies [71]. In our study, we used a decision tree model to identify the most important environmental variables affecting snail distribution in the rivers of Shenzhen and adjacent areas in China. The kappa (K) values show that the models had fair to moderate predictive performance, indicating that certain snail species have clear environmental requirements within the studied habitat gradient. The results show that the occurrence of predators and competitors, canopy cover, chlorophyll-*a*, pollution, substrates, water depth, and velocity are the main variables by which to determine the abundance and distribution of snail intermediate hosts of parasites. Moreover, the canonical correspondence analysis (CCA) obtained similar results.

Our results indicate that biological factors such as predators and competitors should be given priority in terms of snail occurrence and abundance; these may inhibit snail populations through predation and competition [49]. Younes et al. [44] pointed out that the density of snails is related to the predation activities of their predators. Several studies have suggested that benthic invertebrates belonging to the orders Coleoptera, Diptera, Odonata, Hirudata, and Hemiptera play a role in significantly reducing populations and could be considered in snail control strategies [44,46]. During the snail sampling, we noted large numbers of fish around some sample sites; however, their species, quantity, and size have not been surveyed in detail. Many studies [72,73,74,75,76] have suggested that fish predators can dramatically alter the population dynamics and diversity of snail species; this should be given more attention in future research.

Aquatic macrophyte cover was another important factor affecting the distribution of freshwater snails [77,78]. It was found that macrophyte coverage had an important influence on snail occurrence and abundance in our study. Abundant macrophytes could provide sufficient food and spawning sites for snails. Many snails were omnivorous species that could feed on large numbers of aquatic plants, and their growth rate is related to the abundance of plants on which they feed [79]. Moreover, macrophytes could provide a refuge for snails to avoid predation by fish and other large animals, as well as the adverse effects of the current and wind [80]. In addition, macrophytes produce dissolved oxygen through photosynthesis and could create better habitat conditions for aquatic macroinvertebrates [81,82].

Chlorophyll-*a* is an important index of phytoplankton biomass, and its content could reflect the nutritional status of the water body, which is a key parameter for water environment research [83]. We observed that the concentration of chlorophyll-*a* was between 0.52 and 31.43 μg/L, with an average value of 6.16 μg/L. Phytoplankton play an important role in snails’ diets. Our study shows that chlorophyll-*a* in water has an important impact on snail occurrence and abundance. A high concentration of chlorophyll-*a* indicated high phytoplankton content in the water column, which could provide sufficient food for the growth and development of snails. For some snail species, such as *B. straminea* and *P. acuta*, these snails were present in large numbers at suitable concentrations of chlorophyll-*a*, even in the absence of macrophyte.

As shown in Figure 7, the occurrence of most snail species is strongly correlated with human disturbance factors such as sewage discharge. Shenzhen and its neighboring areas are economically developed and densely populated [84], with a huge amount of daily sewage discharge, and some sewage may be directly discharged into rivers without treatment [85]. In addition, the reclaimed water treated by wastewater treatment plants is still high in nutrient content. Human disturbance, especially the high concentration of organic matter and dissolved ions in sewage discharge, provides abundant nutrients for phytoplankton and algae, which increases the content of chlorophyll-*a* in the water and provides sufficient food for snails. Moreover, the ions in wastewater discharge, such as calcium ions, are also a key component in snail shell growth and development [86]. These factors contribute to the presence of snail species in wastewater discharge, which has been confirmed by previous reports [18,87,88]. *Biomphalaria* spp., belonging to Pulmonata, are better adapted to harsher environmental conditions because they can absorb atmospheric air through a vascularized mantle cavity [30]. Since most other freshwater vertebrate and invertebrate fauna cannot cope with low oxygen levels, air-breathing snails are released from competition and predation pressures in hypoxic habitats, which increases their probability of occurrence and abundance [49]. A study by Pinto et al. [89] showed that the discharge of untreated sewage has brought about algal blooms and aquatic macrophyte proliferation in the Pampulha reservoir in Brazil, which contributes to the establishment of high densities of snails in the water body.

In our study, it was found that the river substrate type was one of the key factors affecting the species and abundance of snails. In the investigated rivers, the species and number of snails were the largest in mixed pebbles and gravels. This may have been due to the heterogeneity of the riverbed substrate, which generated the diversity of the spatial distribution of water flow and nutrients and contributed to a plentiful habitat environment [90], thus increasing the occurrence of freshwater snails. In addition, our survey also found that there were fewer snails in sandy environments. This was mainly because, under the same water flow conditions, sand was more easily disturbed than gravel, pebbles, and silt, and had poor stability, which causes severe disturbance to the living environments of benthic invertebrates [91]. Our findings are consistent with the observations of Jowett [92], who found that benthic macroinvertebrate assemblages are greatly dependent on the streambed stability at the reach scale.

Water depth and velocity are also key variables as determinants of snail occurrence. Beisel et al. [93] pointed out that, in addition to the substrate, the water depth and velocity are relatively more important physical factors affecting the community structure of benthic invertebrates. Our observations indicate that almost all the snails in this area prefer to live in shallow water, and it was difficult to find traces of them if the depth was over 40 cm. The shallow water was generally rich in aquatic plants, phytoplankton, and organic matter, which provide abundant food for freshwater snails. However, Mandahl-Barth [94] demonstrated that *Biomphalaria smithi* could be found at a depth of 4.3 m in Lake Edward, Uganda, and *Biomphalaria choanomphala* at 12.2 m in Lake Victoria, Uganda. Freitas [95] observed *Biomphalaria glabrata* survival at a depth of 4~5 m at the bottom of the Lagoa Santa in Brazil. In our study, it was difficult to determine whether the water depth affected the snail distribution and to what extent, although they appear to prefer shallow waters [18]. Thus, in future studies, further efforts should be devoted to determining the quantitative relationship between the distribution of snails and water depth in Shenzhen and adjacent areas to better understand the snail ecology. Snails often inhabit marshes, puddles, canals, ponds, and rivers with slowly running or stagnant water. Fast-running water appears to hinder their predation and the establishment of breeding colonies of snails [96]. Moreover, an excessive current flow would directly flush away the snails and reduce the abundance of the snail species; these phenomena were commonly observed during the investigation in the wet season.

In this study, snails tended to occur at high frequency and were abundant in water bodies with high human activity, such as sewage discharge. The high concentration of organic matter and ions in these polluted waters provided favorable conditions for snail growth and propagation. Our observations also indicated snails with fewer species and smaller numbers in clean water, which were less affected by anthropogenic disturbances. These water bodies host various predators and competitors of invertebrates, such as Coleoptera, Odonata, Hirudinae, and Hemiptera, whose presence significantly inhibits snail density [44]. Several studies have shown that these invertebrate assemblages are responsible for a significant reduction in snail populations that could be considered in integrated snail control measures [46]. Therefore, comprehensive snail control strategies should give priority to reducing the occurrence and abundance of hosts among freshwater snails in order to control the spread of snail-borne diseases at the local scale. This suggests that the proper management of water bodies to reduce water pollution may be one of the most suitable strategies for the comprehensive control of snail-borne diseases in Shenzhen and adjacent areas.

## 5. Conclusions

In this study, a total of nine species of snail were collected throughout Shenzhen and the adjacent region. It was found that *Biomphalaria straminea* (31.4%) was the most abundant snail species, followed by *Sinotaia quadrata* (25.5%) and *Physella acuta* (22.5%). Decision tree models and canonical correspondence analysis showed that the water quality, physical habitat characteristics, and biotic factors were found to be the main variables determining the occurrence and abundance of snail species in the study area. This study also revealed that water bodies disturbed by human activities such as sewage discharge are more likely to host snails and in larger numbers than undisturbed waters, as more snail predators and competitors are present in clean water. Thus, it is recommended to reduce the water pollution in river ecosystems, given that the water pollution is conducive to the presence of snails. The findings of this study could be helpful to inform effective interventions to prevent and control snail-borne diseases in this area.

## Figures and Tables

**Table 1:** Input variables used for model development: minimum values, maximum values, mean values, and standard deviations.

Variable	Unit	Min.	Max.	Mean	SD
Water temperature	°C	20.5	33	26.33	2.78
River depth	Meter	0.03	1.78	0.39	0.34
Water velocity	m/s	0	0.7	0.22	0.16
River width	Meter	3	620.0	63.03	121.90
Dissolved oxygen	mg/L	2.67	17.11	7.86	3.32
pH	-	6.4	8.56	7.48	0.39
Total dissolved solids	mg/L	60.45	4394.5	418.0	558.7
Electrical conductivity	μS/cm	6.8	6754	618.7	902.3
Total nitrogen	mg/L	0.64	27.5	9.75	6.65
Nitrate and nitrites	mg/L	0.1	23.6	4.49	4.45
Ammoniacal nitrogen	mg/L	0.02	14.89	3.72	4.05
Total phosphorus	mg/L	0.02	2.97	0.61	0.66
Orthophosphate	mg/L	0	1.9	0.36	0.47
Chlorophyll-*a*	μg/L	0.52	31.43	6.16	5.26
Chemical oxygen demand	mg/L	0.55	8.7	3.90	1.87
Emergent macrophyte width	Meter	0	30	3.26	5.40
Floating macrophyte width	Meter	0	100	2.36	11.79
Emergent macrophyte cover	Very low(0), Low(1), Moderate(2), High(3)	NA	NA	NA	NA
Floating macrophyte cover	Very low(0), Low(1), Moderate(2), High(3)	NA	NA	NA	NA
Submerged macrophyte cover	Very low(0), Low(1), Moderate(2), High(3)	NA	NA	NA	NA
Substrate type	Silt(0), Sand(1), Gravel(2), Pebble(3), Cobble(4), Boulder(5)	NA	NA	NA	NA
Predator occurrence	Absent(0), Present(1)	NA	NA	NA	NA
Competitor occurrence	Absent(0), Present(1)	NA	NA	NA	NA
Predator abundance	Count	0	504	15	57
Competitor abundance	Count	0	672	43	115
Fishing	Absent(0), Minimal(1), Medium(2), High(3)	NA	NA	NA	NA
Shipping	Absent(0), Minimal(1), Medium(2), High(3)	NA	NA	NA	NA
Clothes washing	Absent(0), Minimal(1), Medium(2), High(3)	NA	NA	NA	NA
Dredging	Absent(0), Minimal(1), Medium(2), High(3)	NA	NA	NA	NA
Pollution discharge	Absent(0), Minimal(1), Medium(2), High(3)	NA	NA	NA	NA
Farming	Absent(0), Minimal(1), Medium(2), High(3)	NA	NA	NA	NA
Irrigation	Absent(0), Minimal(1), Medium(2), High(3)	NA	NA	NA	NA

**Table 2:** Freshwater gastropod species found in the study area that can transmit parasitic diseases and the parasites they can carry.

Family	Genus	Species	Parasites	Reference
Viviparidae	*Sinotaia*	*Sinotaia quadrata*	*A. cantonensis*	[61]
		*Sinotaia limnophila*	*E. revolutum*	[62]
	*Cipangopaludina*	*Cipangopaludina chinensis*	*A. cantonensis*	[61]
Physidae	*Physella*	*Physella acuta*	*A. cantonensis*	[61]
Lymnaeidae	*Radix*	*Radix auricularia*	*A. cantonensis*; *F. hepatica*	[63]
Planorbidae	*Biomphalaria*	*Biomphalaria straminea*	*S. mansoni*; *A. cantonensis*	[64,65]
Semisulcospiridae	*Semisulcospira*	*Semisulcospira cancellata*	*A. cantonensis*; *C. sinensis*; *P. westermani*	[66]
		*Semisulcospira libertina*	*Paragonimus westermani*	[67]
Ampullariidae	*Pomacea*	*Pomacea canaliculata*	*A. cantonensis*; *E. revolutum*; *G. spinigerum*	[68,69,70]

**Table 3:** Occurrence and abundance of freshwater snail species at 102 sampling stations in the study area. All collected snails are intermediate hosts of parasites. The frequency of occurrence is the proportion of the number of sampling sites where the snails appear to the total number of sampling sites (n = 102).

Species	Number of Collected Snails	Percentage of Total Snail Number	Number of Sites	Frequency of Occurrence
*Biomphalaria straminea*	2865	54.7%	32	31.4%
*Sinotaia quadrata*	679	13.0%	26	25.5%
*Physella acuta*	448	8.6%	23	22.5%
*Semisulcospira cancellata*	496	9.5%	15	14.7%
*Radix auricularia*	232	4.4%	14	13.7%
*Semisulcospira libertina*	409	7.8%	12	11.8%
*Pomacea canaliculata*	32	0.6%	9	8.8%
*Sinotaia limnophila*	29	0.6%	7	6.9%
*Cipangopaludina chinensis*	48	0.9%	4	3.9%

## Data Availability

Not applicable.

## Supplementary Materials

The following supporting information can be downloaded at: https://www.mdpi.com/article/10.3390/tropicalmed7120426/s1, Table S1: Investigated sites are shown (XLSX); Figures S1–S3: Overview of importance of the different input variables used in the decision tree models to model the presence or absence of *Biomphalaria straminea*, *Sinotaia quadrata* and *Physella acuta*, respectively (DOCX).
