# Supplementary material for: Environmental Factors Affecting Freshwater Snail Intermediate Hosts in Shenzhen and Adjacent Region, South China
Source: Trop Med Infect Dis. 2022 Dec 9;7(12):426. doi: 10.3390/tropicalmed7120426 (PMC9782201; doi:10.3390/tropicalmed7120426)
Supplement: Supplementary file 1 [file tropicalmed-07-00426-s001.zip › tropicalmed-2080375-supplementary.pdf]

Supplementary Materials: Overview of importance of the different input variables used in the decision tree models to model the presence or absence of *Biomphalaria straminea*, *Sinotaia quadrata* and *Physella acuta*, respectively (DOCX)

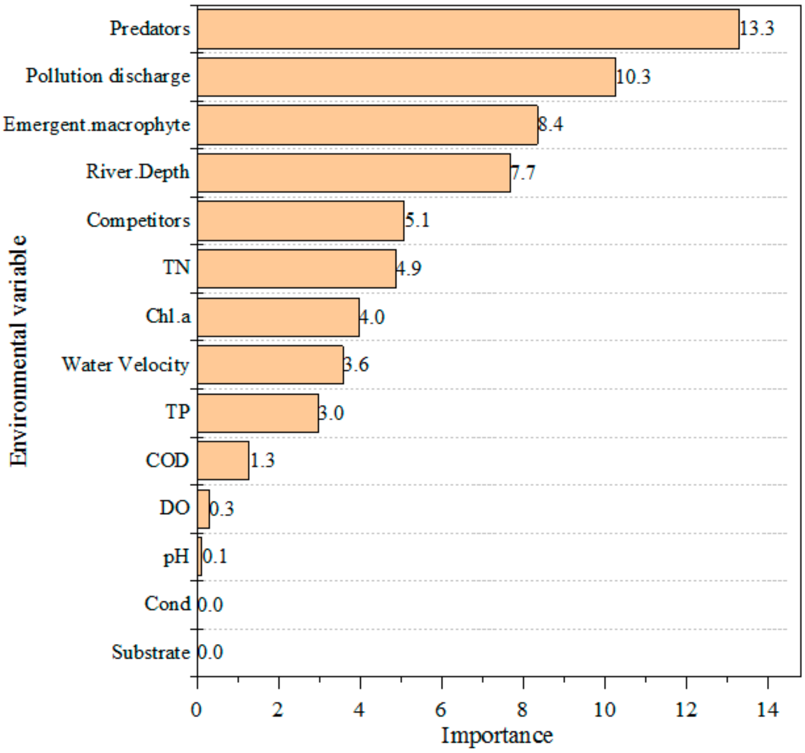

Figure S1. Overview of importance of the different input variables used in the decision tree models to model the presence or absence of *Biomphalaria straminea*.

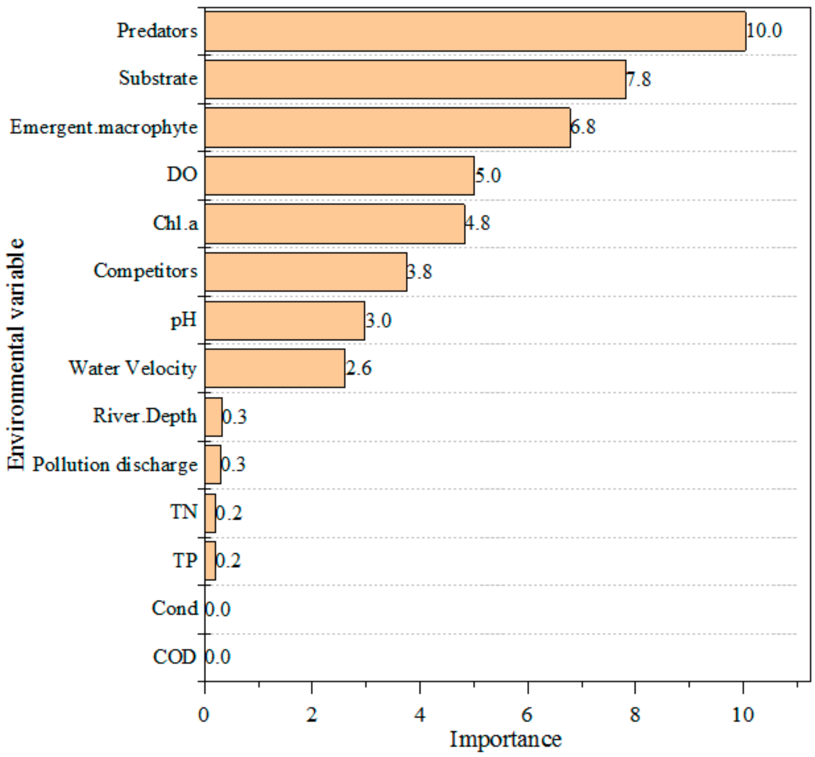

Figure S2. Overview of importance of the different input variables used in the decision tree models to model the presence or absence of *Sinotaia quadrata.*

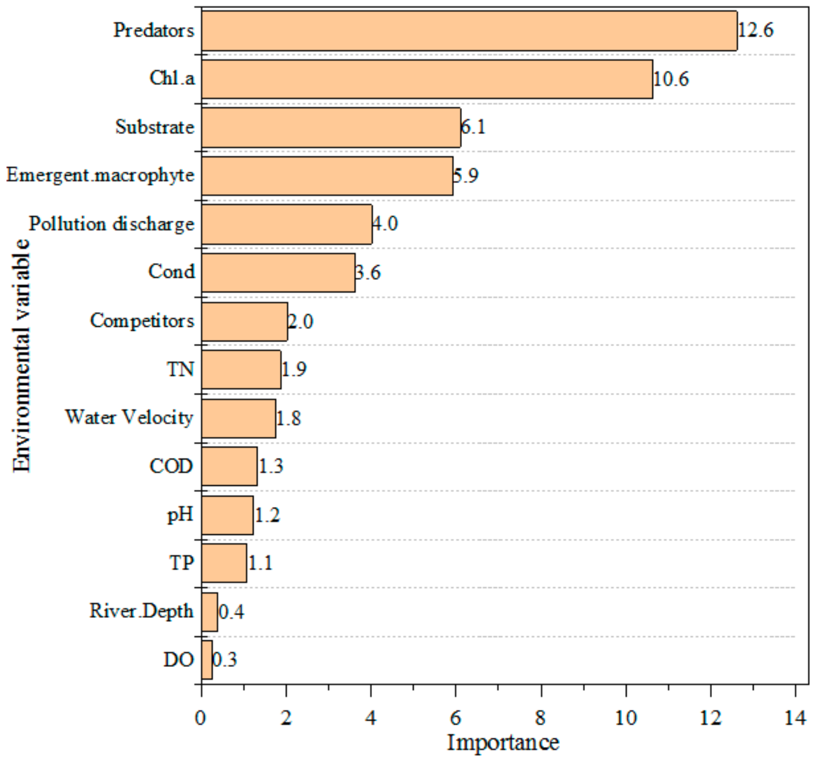

Figure S3. Overview of importance of the different input variables used in the decision tree models to model the presence or absence of *Physella acuta.*
